# Insecticide Exposure Triggers a Modulated Expression of ABC Transporter Genes in Larvae of *Anopheles gambiae* s.s.

**DOI:** 10.3390/insects10030066

**Published:** 2019-03-05

**Authors:** Valentina Mastrantonio, Marco Ferrari, Agata Negri, Tommaso Sturmo, Guido Favia, Daniele Porretta, Sara Epis, Sandra Urbanelli

**Affiliations:** 1 Department of Environmental Biology, Sapienza University of Rome, 00185 Rome, Italy; valentina.mastrantonio@uniroma1.it (V.M.); agata.negri@uniroma1.it (A.N.); mattomas@live.it (T.S.); daniele.porretta@uniroma1.it (D.P.); sandra.urbanelli@uniroma1.it (S.U.); 2 Department of Biosciences and Pediatric Clinical Research Center, University of Milan, 20133 Milan, Italy; mferrari@txbiomed.org; 3 Texas Biomedical Research Institute, San Antonio, TX 78227, USA; 4 School of Bioscience and Veterinary Medicine, University of Camerino, 62032 Macerata, Italy; guido.favia@unicam.it

**Keywords:** insecticide stress, mosquitoes, vector-control, chemical defensome, ABC transporters, pyrethroids

## Abstract

Insecticides remain a main tool for the control of arthropod vectors. The urgency to prevent the insurgence of insecticide resistance and the perspective to find new target sites, for the development of novel molecules, are fuelling the study of the molecular mechanisms involved in insect defence against xenobiotic compounds. In this study, we have investigated if ATP-binding cassette (ABC) transporters, a major component of the defensome machinery, are involved in defence against the insecticide permethrin, in susceptible larvae of the malaria vector *Anopheles gambiae* sensu stricto. Bioassays were performed with permethrin alone, or in combination with an ABC transporter inhibitor. Then we have investigated the expression profiles of five ABC transporter genes at different time points following permethrin exposure, to assess their expression patterns across time. The inhibition of ABC transporters increased the larval mortality by about 15-fold. Likewise, three genes were up-regulated after exposure to permethrin, showing different patterns of expression across the 48 h. Our results provide the first evidences of ABC transporters involvement in defence against a toxic in larvae of *An. gambiae* s.s. and show that the gene expression response is modulated across time, being continuous, but stronger at the earliest and latest times after exposure.

## 1. Introduction

Vector-borne diseases, caused by pathogens and parasites transmitted by bloodsucking arthropods, such as mosquitoes, sandflies, ticks, and tse-tse flies, are a major threat to human health and well-being [1]. Among them, malaria, transmitted by *Anopheles* mosquitoes, is undoubtedly one of the most serious life-threating diseases for humans [1]. Vector control through insecticides remains a main tool for malaria prevention, although progress toward the development of alternative or complementary vector control strategies has been made [2,3]. However, environmental pollution and the increasing insecticide resistance are seriously hampering the use of insecticides and their efficacy. Resistant mosquitoes, belonging to different species, have indeed been recorded in 64 countries with ongoing malaria transmission, with resistance to pyrethroids, being the most common [4,5,6].

Chemical detoxification is likely to play an important role in the development of insecticide resistance. Detoxification in insects is achieved through an array of protein systems, including detoxifying enzymes and efflux pump transporters [7,8,9,10,11]. Natural selection can act on the genes coding for detoxifying proteins, promoting the evolution of metabolic resistance. In susceptible strains, the assessment of the expression of detoxifying genes in response to insecticides could contribute to the comprehension of the molecular mechanisms underlying insecticide detoxification. Furthermore, detoxifying genes could be exploited as targets for novel classes of insecticide compounds. For example, the inhibition of detoxifying efflux pumps has been shown to increase the susceptibility to insecticides in several arthropod species, including malaria vectors [7,8,9,10]. Combined treatments of insecticides with inhibitors of efflux pump transporters could therefore allow to reduce the dose and frequency of insecticide applications and to generate a cascade of positive effects (e.g., reduction of the risk of resistance development, minor pesticide pollution and reduced damage to non-target fauna) [12,13]. In this context, a major goal is to identify the genes encoding for the detoxifying proteins in order to achieve species- and gene-specific inhibition [7,8,9,10].

ATP-binding cassette (ABC) transporters are ATP-dependent efflux pumps, located in the cellular membrane of prokaryotic and eukaryotic organisms, belonging to the ATP-binding cassette (ABC) transporter family. Among the eight sub-families (i.e., ABCB-ABCH) included in the ABC family, three of them, ABCB, ABCC and ABCG, are involved in xenobiotic detoxification. ABC transporters are part of a wider defence system comprising several gene families and pathways that allow an organism to transform and eliminate toxic chemicals (chemical defensome) [12,13,14,15,16,17,18,19].

In recent years, several studies have documented that ABC transporters play an important role in insecticide detoxification in a variety of arthropod species, ranging from ticks, to bed bugs, to body lice and mosquitoes [9,13,19,20,21,22,23,24,25]. On the other hand, the pattern of ABC transporter involvement in insecticide detoxification is different in different strains (resistant vs. susceptible), life-stages (adults vs. larvae) and sexes of the same species [8,26,27]. In addition, even though ABC transporters can act against all major insecticide classes, including organochlorines, organophosphates, pyrethroids and even *Bacillus thuringensis* (Bt) toxins, some heterogeneity can be observed within and between species [14,28]. For example in the mosquito *Culex pipiens*, ABC transporters have been shown to be involved against endosulfan, ivermectin and cypermethrin, but not against chlorpyrifos [24]. Likewise, ABC transporters were shown to be involved against temephos in the mosquitoes *Aedes caspius* [13] and *Ae. aegypti* [10], but not in *Anopheles stephensi* [15]. Further studies are thus required to determine the occurrence of common patterns in ABC transporter engagement, in relation with taxa or insecticide classes.

This study is focused on the mosquito *Anopheles gambiae* sensu stricto, the principal malaria vector in sub-Saharan African regions, and on the pyrethroid insecticide permethrin. Pyrethroids, among the different classes of insecticides recommended by the World Health Organization (WHO), still occupy a prominent position in malaria vector control. They are indeed widely used on approved long-lasting insecticidal nets (LLINs) and indoor residual spraying programmes (IRS) to control adult mosquitoes [1].

In the case of *An. gambiae* s.s., there are circumstantial evidences for the involvement of ABC transporters in pyrethroid resistance, i.e., the results from a whole genome-transcriptomic study, showing a constitutive over-expression of one ABCC member in adult deltamethrin-resistant individuals [29]. More recently, ABC transporters have been shown to be involved in the resistance against pyrethroids in adult individuals [20]. In addition, due to the wide use of pyrethroids in crop pest control, also larval stages of *An. gambiae* can be exposed to these insecticides in the developing sites near crop fields, which would lead to a further risk of insecticide resistance insurgence [30,31,32]. To date, however, no studies have been focused on the role of ABC transporters against pyrethroids in larval stages of this species. Here, we investigated the potential association between ABC transporters and permethrin using both laboratory bioassays and gene induction experiments. We conducted laboratory bioassays with permethrin alone or in combination with an ABC transporters inhibitor, to assess if these efflux pumps are a mechanism of defence against this insecticide in the larval stage. In the genome of *An. gambiae*, 34 ABC transporter genes have been identified [14]. Among them, we investigated the expression profiles of five genes encoding for ABC transporters that were previously found involved in insecticide detoxification in other arthropod species [10,33,34,35,36,37,38].

## 2. Materials and Methods

### 2.1. Mosquitoes

All mosquito larvae used in this study derived from an *An. gambiae* s.s. colony from the University of Camerino (Camerino, Italy), previously tested for its susceptibility to permethrin (2 < ratio RR50 < 5, according to Fossog and coworkers [39]). This colony was obtained in 2008 from the Centre National de Recherche et de Formation sur le Paludisme (CNRFP) in Ouagadougou (Burkina Faso, West Africa), and descended from wild mosquitoes collected in Burkina Faso. Mosquitoes have then been reared for about 9 years in the Camerino insectary, where they were maintained at standard lighting conditions of 12 h light and 12 h dark, a relative humidity of 80 ± 5% and temperature of 30 °C, in aseptic conditions during both the immature and adult stages. The larvae were grown in tanks filled with culture water containing sterile minced commercial mouse food.

### 2.2. Bioassays

In order to assess if ABC transporters are involved against permethrin in larvae of *An. gambiae* s.s., bioassays with insecticide, alone or in combination with a sub-lethal dose of the ABC inhibitor verapamil, were carried out, according to standard protocols [32]. Groups of 25 third-instar larvae were placed into 250 mL plastic cups with 100 mL of spring water, and then insecticide or insecticide plus inhibitor were added. In the bioassays with insecticide alone, six concentrations of permethrin (PESTANAL, Sigma-Aldrich S.r.l., Milan, Italy) were used, ranging from 15 to 700 ppb (i.e., 15, 47, 92, 230, 350 and 700 ppb), leading to a mortality from 1 to 99%. In the bioassays with insecticide and insecticide plus verapamil, four concentrations were tested: 4.8, 15, 47 and 92 ppb. To determinate the sub-lethal dose of verapamil (i.e., the highest dose at which no dead larvae were observed) six concentrations were used (20, 40, 80, 100, 160 and 240 ppm). Larvae treated only with acetone (the solvent of permethrin, Sigma-Aldrich S.r.l., Milan, Italy) and water were also included as controls. All experiments were performed in quadruplicate. Mortality data were registered at 24 h after treatment and analysed by Probit regression analysis to estimate LD_50_ dose and their 95% confidence intervals (CIs) using the Poloplus software [40].

The synergistic ratio was then calculated from the LD_50_ doses estimated from the treatments using permethrin and permethrin plus verapamil to assess the effect of the ABC transporters inhibition on the *An. gambiae* s.s. larvae. The hypotheses of equality (equal slopes and intercepts) and parallelism (equal slopes) of the regression lines of the two treatments were also tested using the likelihood-ratio test, as implemented in the software Poloplus.

### 2.3. Induction of ABC Transporter Gene Expression

Five genes encoding for ABC transporters were analyzed for their expression profiles in treated or untreated larvae of *An. gambiae* s.s: three members of the ABCB sub-family (i.e., AGAP005639, AGAP006273, AGAP002278); one member of the ABCC sub-family (AGAP006427); and one member of sub-family ABCG (AGAP001333). They were selected in order to investigate members of all ABC transporter sub-families involved in insecticide defence (ABCB, ABCC and ABCG), and on the basis of previous studies showing their involvement against insecticides in susceptible and resistant insect species [10,33,34,35,36,37,38].

Oligonucleotide primers were designed from the sequences of each gene using the Primer3, Beacon Designer^TM^ and mFold softwares [41] and used for standard and quantitative RT-PCRs. In order to confirm the specificity of the amplifications, the amplicons obtained using standard PCR conditions (see below) were sequenced and the obtained sequences blasted into the Genbank database.

In order to analyze the expression profile of ABC transporter genes after permethrin exposure, mosquito larvae were treated with the insecticide following the protocol described above. The LD_50_ dose of permethrin (297.84 ppb), estimated by the above bioassays, was used to treat the larvae; larvae at five time-points after exposure (2, 4, 6, 24 and 48 h) were collected and analyzed. Six replicas were effected for each time point. Three pools of 10 individuals, each pool from two plastic vessels, were collected at the five time-points, and stored in RNA later at −80 °C until subsequent molecular analysis. Negative controls (larvae treated only with acetone and water), were also collected for each time of exposure. RNA was extracted from each pool of individuals using the RNeasy Mini Kit (Qiagen, Hilden, Germany), according to the manufacturer’s instructions. Then, total RNA was eluted into nuclease-free water and the concentration of RNA was determined at 260 nm using a NanoDrop ND-2000 (Thermo Fisher Scientific, Waltham, MA, USA). cDNAs were synthesized from 150 ng of total RNA using a QuantiTect Reverse Transcription Kit (Qiagen, Hilden, Germany) with random hexamers. The cDNA was used as template in reverse transcription (RT)-PCR reactions using the primers reported in Table 1.

RT-PCRs on target genes were performed using a BioRad CFX Real-Time PCR Detection System (Bio-Rad, Hercules, CA, USA) with the following conditions: 50 ng of cDNA; 250 nM of forward and reverse primers; 98 °C for 30 s, 40 cycles of 98 °C for 15 sec, 57–59 °C for 30 sec; fluorescence acquisition at the end of each cycle; melting curve analysis after the last cycle. In order to analyze the expression of the target genes, the efficiency of the new primer pairs was assessed trough the analysis of the standard curves, then the cycle threshold (Ct) values were determined for each gene and normalized according to two endogenous reference genes: the ribosomal protein S7 (*rps7*) gene and the actin gene (*act 5C*) (Table 1). The expression of ABC transporters genes in the control group was considered as the basal level. Shapiro–Wilk test was performed to check normality of the normalized expression data. For each gene, univariate two-way ANOVA was performed to compare differences in relative expression between treated and control larvae and among time points. Subsequent post-hoc Tukey tests were performed for pairwise comparisons among time points. All analyses were performed using the software IBM SPSS Statistics (IBM Corp. Released 2013, Armonk, NY, USA).

## 3. Results

### 3.1. Laboratory Bioassays

The sub-lethal dose of the inhibitor verapamil was 80 ppm, as no mortality was observed at this concentration, while dead larvae were observed from the 100 ppm concentration (Figure S1). Mortality data of the bioassays with insecticide and insecticide plus verapamil recorded at 24 h were well described by the probit dose–response model (chi-squared goodness-of-fit test, *p* > 0.05). Both the hypotheses of the regression line equality and parallelism between treatments were rejected, showing significant differences between the two treatments (Table 2).

The LD_50_ dose and 95% Confidence Intervals (CIs) estimated was 297.84 ppb (248.5–368.18) for the treatment with permethrin alone, and 18.69 ppb (15.89–21.81) for treatment with insecticide plus verapamil. A synergistic factor of 15.94 (12.37–20.53) was estimated between the LD_50_ of the two treatments (Table 2, near here).

### 3.2. Expression Profiles of ABC Transporter Genes after Permethrin Exposure

The sequencing of the amplicons obtained by conventional PCRs confirmed the specificity of the amplification for each selected ABC transporter gene, with an identity of 100% with the sequences of the ABC transporter genes of *An. gambiae* s.s. available in the databases.

**Figure 1:**
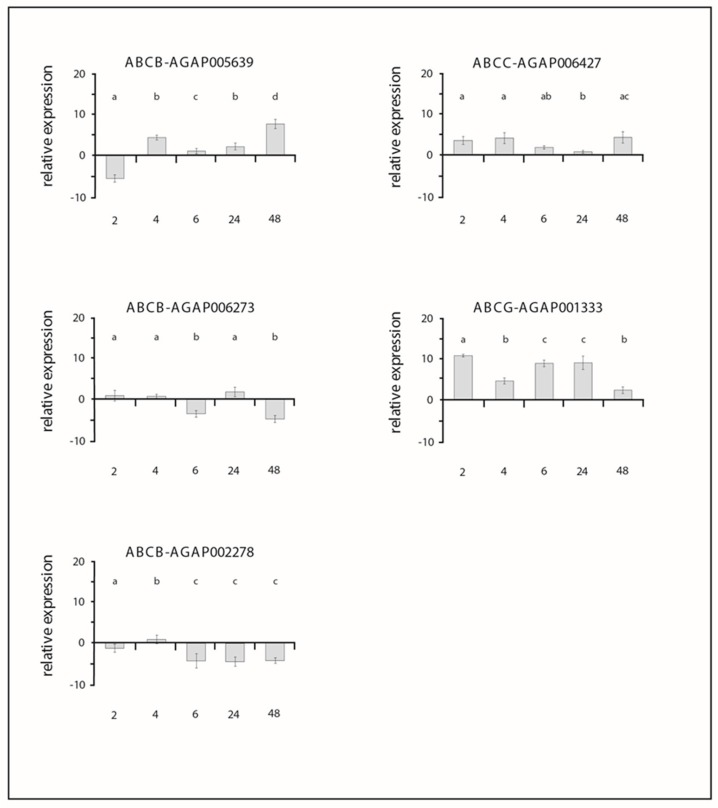
Relative expression of *Anopheles gambiae* s.s. ABC genes measured by quantitative PCR after different times of permethrin exposure. The expression level in non-treated larvae was considered to be the basal level. The internal reference genes *rps7* and *act5C* for *An. gambiae* s.s. were used to normalize the expression levels. The values are expressed as means ± standard deviations. In x-axes: time of permethrin exposure: 2, 4, 6, 24 and 48 h. For each gene, equal letter means post-hoc Tukey tests *p* > 0.05; different letter means post-hoc Tukey tests *p* < 0.05.

All primer pairs used to amplify ABC transporter genes showed an efficiency ranging from 95% to 105%. The results obtained from gene expression analyses for each gene in treated larvae, in comparison with the untreated larvae, are shown in Figure 1 and Table S1.

The Shapiro–Wilk test showed normal distribution of the data (all tests *p* > 0.05). The ANOVA analysis showed that the expression levels of all ABC genes analysed were significantly affected by permethrin treatment, by the time of larval exposure and by the combination of these two factors (Table S2). For each gene, post-hoc Tukey tests showed significant differences among the expression levels across time (Figure 1).

## 4. Discussion

### 4.1. Involvement of ABC Transporters in Permethrin Detoxification

The use of ABC transporter inhibitors has greatly contributed to document the involvement of these efflux pumps in defence/resistance against insecticides in arthropod vectors [14]. More recently, transcriptional studies allowed not only to further support the detoxifying role of ABC transporters, but also to identify which genes encoding for ABC transporters are mainly involved [12,14,15]. In this study, using both of the above experimental approaches, we have provided evidence for the involvement of ABC transporters in the defence against permethrin in larvae of *An. gambiae* s.s. By exposing third-instar larvae to the LD_50_ dose of permethrin, indeed, we observed that mortality was about 15-fold greater after the treatment with insecticide plus ABC inhibitor, than after treatment with insecticide alone (Table 2). Likewise, the exposure of larvae to the insecticide led to transcriptional induction of three out of the five ABC transporter genes analyzed (Figure 1), which further supports the involvement of ABC transporters in the defence against permethrin [14].

Among the ABC transporter genes analyzed in *An. gambiae* s.s., some of them showed an activation pattern similar to that observed in other insect species. For example, genes orthologues to the ABCG-AGAP001333 gene here examined have been shown to be over-transcribed in *Anopheles arabiensis* and in *An. stephensi* mosquitoes exposed to Dichlorodiphenyltrichloroethane (DDT) and permethrin, respectively [19,33,34,35]. These results indicate that this ABCG transporter is likely to play an important role in different mosquito species (as well as in other insects, e.g., in *Bemisia tabaci* exposed to neonicotenoids [38]) as a defence system against different insecticides. Notably, the *Anst*ABCG4 transporter gene (ASTE008861) of *An. stephensi* was found over-expressed after 2, 4, 6, 24 and 48 h of exposure to the LD_50_ dose of permethrin [34], which is the same pattern that we observed in *An. gambiae* s.s. larvae. The comparison of the expression profiles in *An. gambiae* s.s. and *An. stephensi* susceptible larvae, exposed to LD_50_ permethrin dose, not only suggests that the ABCG4 transporter is involved in defence against permethrin in both species, but also shows that the induction pattern is similar. Similarity between the two species can also be observed in the transcriptional response of the orthologous genes ABCB-AGAP006273 and ASTE000608/*Anst*ABCB3: in the larvae of both species, no-differential expression or up-regulation were observed during the 2–48 h of permethrin exposure.

The other ABC transporters analysed in *An. gambiae* s.s. showed a more heterogeneous involvement among species and insecticides. This is the case of the ABCB-AGAP002278 gene that showed no transcriptional induction by permethrin in *An. gambiae* s.s. larvae (Figure 1), while its orthologous in *An. stephensi* (ASTE003466/*Anst*ABCBmember6) was found up-regulated in larvae exposed to this insecticide [33,34]. Similarly, the ABCB-AGAP005639 gene, that we found up-regulated in *An. gambiae* s.s. after 4 and 48 h of exposure, was found down-regulated in previous studies on *An. stephensi* (ASTE009548/*Anst*ABCB2) [33,34]. Interestingly, in susceptible larvae of the mosquito *Aedes aegypti*, the transporter *Aaeg*P-gp–AAEL010379-PA, orthologous to the *An. gambiae* s.s. ABCB-AGAP005639, showed an increased expression of about eight-fold compared to control after 48 h of exposure to the LD_50_ dose of the organophosphate temephos, which supports its involvement in temephos defence [10]. In summary, although some similarities exist between species in the response to insecticides, more studies are needed to assess if there are conserved patterns.

Regarding *An. gambiae* s.s, future studies addressing the gene expression of other ABC transporter genes during insecticide exposure are needed to gain a more comprehensive picture of how many ABC transporter genes are involved in the defense against permethrin. Furthermore, mRNA expression studies could also be addressed to analyse the ABC transporter gene expression, not only after the exposure to the toxic, but also after treatment with ABC transporter inhibitors or after treatment with inhibitors in combination with insecticides. For example, the exposure to verapamil of MCF-7 human mammary carcinoma cell lines and hepatocellular carcinoma has been shown to reduce the expression levels the MDR1 ABC transporter gene [42,43]. Likewise, a significant decrease in P-gp expression and transport activity has been observed when L1210/VCR neoplastic cells were treated with transretinoic acid (ATRA) and verapamil [44]. Although the mechanism of down-regulation of ABC transporter genes by verapamil is not known, these data suggest that it might influence the ABC transporters at both protein and mRNA levels. In arthropods, at our best knowledge, verapamil has been used only as synergist and no data are available about the expression levels of ABC transporter genes under verapamil exposure, alone or in combination with an insecticide [14]. If a down-regulation effect of ABC transporter genes by verapamil would exist, as in human cancer cell lines, it could in part explain the lethal effect on exposed larvae to permethrin plus verapamil. Furthermore, the analyses of the expression patterns in larvae exposed to verapamil or in association with permethrin could contribute to describe the possible involvement of ABC transporter genes in cellular defense.

### 4.2. Dynamics of Gene Transcriptional Induction during Permethrin Exposure

The dynamics of the detoxifying gene activation during insecticide exposure is currently poorly investigated in arthropods [19,27,33,34]. Most of the studies that analysed the transcriptional response of defensome genes [19] after insecticide exposure have indeed been focused on a single time point [14]. Some studies that have recently investigated the expression profiles of detoxifying genes at different time points during insecticide exposure, are showing that defence response to insecticide stress is a dynamic process where the genes involved in the cellular defence can turn on and off at different time points during insecticide exposure [19,34,45,46,47,48].

With respect to the ABC transporters, this pattern can be due to the specificity between insecticide substrate and the transporter. Different ABC transporter genes have been indeed shown to be up regulated against different insecticides within the same species [8,15,26,27]. Furthermore, the high cost of detoxification can led to a reallocation of the energetic resources and, consequently, to the turn off of some genes during insecticide exposure. Two out the five ABC transporter genes analysed in *An. gambiae* s.s. were down-regulated or not significantly up-regulated during the permethrin exposure, which supports the above view (Figure 1).

The analysis of gene expression profiles across time allowed us to show not only which genes encoding for ABC transporters were up-regulated, but also the dynamics of their transcriptional response during permethrin exposure. The three genes that were found up-regulated in *An. gambiae* s.s., showed different patterns of expression across the 48 h of permethrin exposure. The ABCB-AGAP005639 was found up-regulated only at 4 and 48 h with a peak of expression registered at 48 h post-exposure (i.e., about eight-fold compared to control). The ABCG-AGAP001333 gene was up-regulated since 2 hours after treatment and its up-regulation persisted until 48 h maintaining similar values across time, similarly to the ABCC-AGAP006427 gene (with the exception of 24 h) (Figure 1). These results showed therefore the occurrence of a modulated response of the ABC transporter genes across time, where each single gene is up- or down-regulated during insecticide exposure at different time-points, and up-regulation of multiple genes occurs at different time-points. By considering the profiles of all up-regulated genes during permethrin exposure, a more general expression pattern can be observed that is consistent with the role of ABC transporters in the Phases 0 and III during the detoxification process [17,18,19]. Consistently, it can be observed that some genes were up-regulated across all time points from 6 to 48 h (ABCC-AGAP006427 and ABCG-AGAP001333) as well as that the ABCB-AGAP005639 gene was up-regulated at early or late time-points (e.g., 4 and 48 h), which depicts a continuous action of ABC transporters across time and would suggest a stronger response at the earliest and latest times after exposure as observed also in the *An. stephensi* mosquito [19].

## 5. Conclusions

The urgency to protect the current chemical weapons used to control arthropod vectors from resistance insurgence and the perspective to find new target sites are fuelling the study of the molecular defence mechanisms against insecticides. In this study, we provide evidences for the involvement of ABC transporter efflux pumps in defence against permethrin in *An. gambiae* s.s. larvae. By analysing the expression profiles of ABC transporter genes at several time points, moreover, we found three genes up-regulated throughout the time of permethrin exposure (48 h). Although exposure time and insecticide dose can affect gene induction, as well as only a subset of the ABC transporter genes has been analysed, our results clearly support the occurrence of a modulated transcriptional response during insecticide exposure in *An. gambiae* s.s. This is consistent with the patterns observed for ABC transporters in the mosquito *An. stephensi* [19,33,34], or in other gene families involved in xenobiotic defence, such as CYP450 [45,46] and Cuticular Proteins (CPs) [48]. Future studies should be directed toward the analysis of induction of all defensome genes to understand how they work together during insecticide stress and to find key genes that could be potential targets for the development of gene-silencing based control tools.

## Figures and Tables

**Table 1:** Primer sequences used to amplify fragments of ATP-binding cassette (ABC) transporters genes in *Anopheles gambiae* s.s.

Vector Base Sequence ID	ABC Sub-Family	Forward 3’-5’	Reverse 3’-5’	PCR Product Size (bp)
AGAP005639	ABCB	TTCATCACGAAACTACCGAAC	GTCCCTTACTTGCTCGCT	204
AGAP006273	ABCB	CACGCTGGGCTATCAGTA	AAAACTTCCACCAATCGAAACG	118
AGAP002278	ABCB	AAAGGTGACAGAGAGGTGTAGGAAA	ACGCCATGCACTAAACTATCACATT	104
AGAP006427	ABCC	AAAGTGTTCTACGGCATGGTGAAG	CAGCCTCCTTAATCGGTTTCAGTTT	108
AGAP001333	ABCG	GTCTCCTGTCGTTGTAGTTTT	CGTAACAGAAACATCGTCCATT	174
AGAP010592	*rps7*	GGCGATCATCATCTACGTGC	GTAGCTGCTGCAAACTTCGG	459
AGAP000651	*act 5C*	TCTGGCACCACACGTTCTAC	CAGGTAGTCGGTGAGATCGC	313

**Table 2:** Toxicity of insecticide and insecticide + ABC transporters inhibitor. LD_50_, 95% Confidence Intervals (95% CI) and slopes estimated from mortality data by probit analysis are shown. SR, synergistic ratio.

Insecticide	Slope (±SE)	LD_50_ (95% CI)	SR (95% CI)	χ^2^ (df)		
				Goodness-of-Fit	Equality	Parallelism
permethrin	1.514 (0.131)	297.84 ppb (248.5–368.18)		2.467 (4)	317 (2) *	11.40 (1) *
permethrin + verapamil	2.259 (0.181)	18.69 ppb (15.89–21.81)	15.94 (12.37–20.53)	13.37 (5)		

* Chi-Square probability *p* < 0.05.

## Supplementary Materials

The following are available online at https://www.mdpi.com/2075-4450/10/3/66/s1, Figure S1: Mortality rate of *Anopheles gambiae* s.s. larvae treated with verapamil, Table S1: Relative expression of *Anopheles gambiae* s.s. ABC genes measured by quantitative PCR after permethrin exposure at different times, Table S2: Univariate two-way ANOVA analysis on relative gene expression in relation to insecticide treatment and time of exposure for each ABC transporter gene analysed.
