# Supplementary material for: Insecticide Exposure Triggers a Modulated Expression of ABC Transporter Genes in Larvae of Anopheles gambiae s.s
Source: Insects. 2019 Mar 5;10(3):66. doi: 10.3390/insects10030066 (PMC6468849; doi:10.3390/insects10030066)
Supplement: Supplementary file 1 [file insects-10-00066-s001.zip › supply/Table S2.docx]

**Table S2.** Univariate two-way ANOVA analysis on relative gene expression in relation to insecticide treatment and time of exposure for each ABC transporter gene analysed.

|  | **df** | **MS** | **F** | ***p*** |
| --- | --- | --- | --- | --- |
| **AGAP005639** |  |  |  |  |
| Insecticide | 1 | 9896 | 17,399 | 0.000 *** |
| Time | 4 | 34,347 | 60,388 | 0.000 *** |
| Insecticide × Time | 4 | 34,347 | 60,388 | 0.000 *** |
| **AGAP006273** |  |  |  |  |
| Insecticide | 1 | 20.667 | 56.160 | 0.000 *** |
| Time | 4 | 15.109 | 41.058 | 0.000 *** |
| Insecticide × Time | 4 | 15.110 | 41.058 | 0.000 *** |
| **AGAP002278** |  |  |  |  |
| Insecticide | 1 | 92.928 | 147.975 | 0.000 *** |
| Time | 4 | 10.270 | 16.354 | 0.000 *** |
| Insecticide × Time | 4 | 10.270 | 16.354 | 0.000 *** |
| **AGAP006427** |  |  |  |  |
| Insecticide | 1 | 40,368 | 148,412 | 0.000 *** |
| Time | 4 | 2643 | 9717 | 0.000 *** |
| Insecticide × Time | 4 | 2643 | 9717 | 0.000 *** |
| **AGAP001333** |  |  |  |  |
| Insecticide | 1 | 275,427 | 909,000 | 0.000 *** |
| Time | 4 | 17,209 | 56,797 | 0.000 *** |
| Insecticide × Time | 4 | 17,209 | 56,797 | 0.000 *** |

*** *p* < 0.001.
