# Supplementary figures and images for: Insecticide Exposure Triggers a Modulated Expression of ABC Transporter Genes in Larvae of Anopheles gambiae s.s
Source: Insects. 2019 Mar 5;10(3):66. doi: 10.3390/insects10030066 (PMC6468849; doi:10.3390/insects10030066)

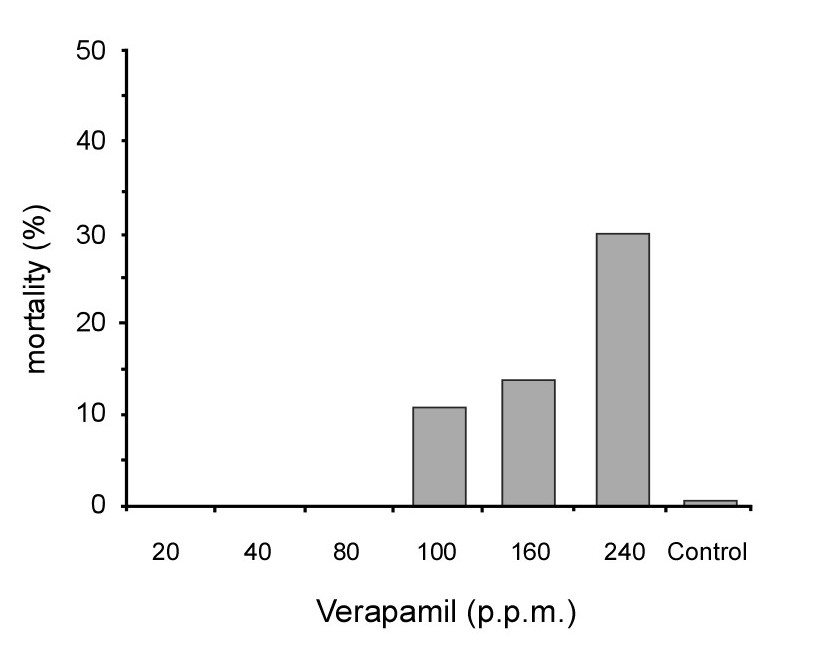


**Figure S1.** Mortality rate of *Anopheles gambiae* s.s. larvae treated with verapamil.

Supplement: Supplementary file 1 [file insects-10-00066-s001.zip › supply/Figure S1.docx]
