# Supplementary material for: Insecticide Exposure Triggers a Modulated Expression of ABC Transporter Genes in Larvae of Anopheles gambiae s.s
Source: Insects. 2019 Mar 5;10(3):66. doi: 10.3390/insects10030066 (PMC6468849; doi:10.3390/insects10030066)
Supplement: Supplementary file 1 [file insects-10-00066-s001.zip › supply/Table S1.doc]

**Table S1.** Relative expression of *Anopheles gambiae s.s.* ABC genes measured by quantitative PCR after permethrin exposure at different times. The expression level in non-treated larvae was considered to be the basal level. The internal reference genes rps7 and *act5C* for An. gambiae s.s. were used to normalize the expression levels. The values are expressed as means ± standard deviations. Times: two, four, six, 24 and 48 hours.

| **Gene ID** | **ABC Sub-family** | **2 h** | **4 h** | **6 h** | **24 h** | **48 h** |
| --- | --- | --- | --- | --- | --- | --- |
| AGAP005639 | ABCB | −5.4 (±1.1) | 4.3 (± 0.6) | 1.6 (± 0.5) | 2.7 (± 1.5) | 7.6 (± 1.2) |
| AGAP006273 | ABCB | 1.3 (±0.7) | 1.3 (± 0.6) | −3.2 (± 0.9) | 2.2 (± 1.1) | −4.9 (± 0.9) |
| AGAP002278 | ABCB | −1.4 (±1.1) | 1.6 (± 1.1) | −4.3 (± 1.6) | −4.3 (± 0.9) | −4.2 (± 0.7) |
| AGAP006427 | ABCC | 3.9 (±0.7) | 4.3 (± 1.1) | 2.5 (± 0.1) | 1.4 (± 0.1) | 4.5 (± 1.0) |
| AGAP001333 | ABCG | 10.9 (±0.1) | 4.4 (± 0.6) | 8.6 (± 0.9) | 8.7 (± 1.1) | 2.7 (± 0.8) |
